# Effects and mechanisms of fermentation media on sensory qualities, nonvolatile components, and microbiota in cigar tobacco leaves

**DOI:** 10.3389/fbioe.2025.1578001

**Published:** 2025-06-17

**Authors:** Qianying Zhang, Jie Liu, Zhishun Chai, Shuanghong Yang, Yun Jia, Hongfei Zhang, Xiaoying Ji, Jinshan Lei, Dongliang Li

**Affiliations:** ^1^ Cigar Fermentation Technology Key Laboratory of China Tobacco (China Tobacco Sichuan Industrial Co., Ltd.), China Tobacco Technology Innovation Center for Cigar, Chengdu, China; ^2^ Industry Efficient Utilization to Domestic Cigar Tobacco Key Laboratory of Sichuan Province, Great Wall Cigar Factory, Shifang, China; ^3^ China National Tobacco Quality Supervision and Test Centre, Zhengzhou, China

**Keywords:** cigar tobacco leaf (CTL), fermentation medium, microbiota, nonvolatile chemical components, amino acid metabolism

## Abstract

**Introduction:**

Medium fermentation is crucial for improving the quality and industrial value of cigar tobacco leaves (CTLs); however, its effect varies by variety. This study examined two CTLs (Dexue No. 1 from Shifang, Sichuan Province, and Chuxue No. 14 from Enshi, Hubei Province) using three fermentation media.

**Methods:**

The effects that different media have on the sensory quality, chemical composition, microbial communities, and metabolism of CTLs were explored to reveal the mechanisms underlying quality differences.

**Results:**

Results indicated that the effect different fermentation media have on the same type of CTL was less pronounced than that which a single medium had on different CTLs. The use of fermentation media altered the nonvolatile components and microbiota of the CTLs. Specifically, fermentation reduced the total polyphenol, neochlorogenic acid, and scopoletin content in Enshi CTLs, but increased the total amino acid and polyphenol content, including threonine, alanine, proline, and cryptochlorogenic acid, in Shifang CTLs. Among the six dominant microbes identified, only *Staphylococcus* was more abundant in Enshi CTLs than in Shifang CTLs, whereas *Ralstonia*, *Pseudomonas*, *Aspergillus*, *Terribacillus*, and *Brachybacterium* exhibited the opposite trend. Fermentation reduced the populations of *R. pseudosolanacearum*, *R. solanacearum*, and *P. psychrotolerans* in Shifang CTLs. A total of 18 genera, including *Staphylococcus* and *Pseudomonas*, contributed to the accumulation of 16 nonvolatile chemical components via carbohydrate and amino acid metabolism. Ultimately, this led to an enhancement in sensory attributes of the CTLs. Analysis of the metabolic network of phenylalanine, proline, and serine further confirmed that *Staphylococcus* played an important role in the metabolism of these three amino acids.

**Conclusion:**

Both fermentation medium and production area characteristics jointly influenced CTLs quality.

## Graphical Abstract

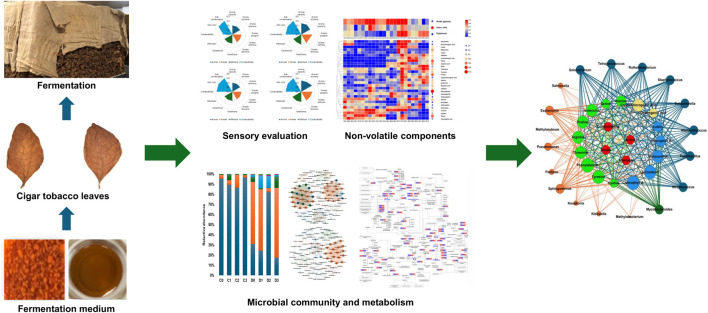

## 1 Introduction

Cigars can be classified into handmade, half machine-rolled, or machine-made types. High-end consumers favor handmade cigars because of their outstanding craftsmanship and quality. The quality of handmade cigars is mainly affected by that of the cigar tobacco leaves (CTLs) used, with geographical origin emerging as a crucial determinant of their quality parameters (Zhao et al., 2024). Current production practices call for the implementation of quality enhancement protocols for moderately inferior CTLs to optimize raw material utilization rates, given that quality variations inherently exist within the supply chain. The appearance and internal quality of CTLs determine their grade and purchase price, making it essential to develop cost-effective and sustainable approaches to improve their availability.

Fermentation is a pivotal process in CTL quality optimization. Recent investigations have demonstrated that supplementation with specific fermentation media can substantially enhance the sensory attributes while modifying the chemical composition, aromatic profile, and microbial community dynamics of CTLs (Zhang et al., 2020). Previous studies have primarily focused on optimizing the fermentation process parameters (Cai et al., 2024; Jia et al., 2023b) and developing new fermentation media, mainly including microorganisms and natural plant extracts (Jia et al., 2023a; Zhang et al., 2023a; Zong et al., 2023; Hu et al., 2024). Research findings have demonstrated that the fermentation media used influences the volatile components, microbial growth, and community succession in CTLs (Wang et al., 2024; Zhang et al., 2024a; Zheng et al., 2022). However, the effects that these media have varies significantly among the different types of CTLs, and the mechanisms underlying these differential effects remain unclear.

Previous studies have demonstrated that three fermentation media—*T. aurantialba* SCT-F3 fermentation broth (Zhang et al., 2023a), cocoa extract (Zong et al., 2023), and a 1:1 composite formulation combining both substrates (Cai et al., 2024)—could enhance the sensory quality of CTLs obtained from Shifang City. The *Tremella aurantialba* SCT-F3 broth is a microbially synthesized fragrance precursor, whereas the cocoa extract constitutes a plant-derived aromatic compound. Shifang (Sichuan Province) and Enshi (Hubei Province) represent the two principal CTL production regions in China, with Shifang boasting over four centuries of cultivation history and Enshi emerging as a significant new production hub. Current Chinese research has predominantly investigated the comparative efficacy that various substrates have on individual cultivars (Ding et al., 2024; Hu et al., 2023; Mao et al., 2023), with limited exploration of plant extract and microbial metabolite interactions across different geographical origins. Furthermore, mechanistic studies elucidating these differential effects are lacking.

In this study, we investigated the effects that three fermentation media have on the quality of CTLs obtained from Shifang and Enshi Cities. Furthermore, we elucidated the fermentation mechanism involved by modeling microbial co-occurrence patterns of the CTLs, analyzing the contributions of nonvolatile chemical components and microbes to sensory evaluation, and reconstructing the key metabolic pathways involved. These findings have significant implications for enhancing quality control and guiding CTL fermentation processes.

## 2 Materials and methods

### 2.1 Fermentation medium preparation

The strain *T. aurantialba* SCT-F3 (CGMCC No.23831) was inoculated into 250-mL flasks with 75 mL of potato glucose broth containing 6 g/L of potato extract powder and 20 g/L of glucose. The cultures were incubated at 28°C under agitation at a speed of 150–180 r/min for a duration of 5 days. After fermentation, the mycelium pellets were removed by centrifugation (5,000 × *g* for 10 min), and the resulting supernatant was utilized as the microbial fermentation medium, following the methodology described by Zhang et al. (2023a). Cocoa extract was obtained by extracting 10.0 g of cocoa powder with sterile pure water in a volume ratio of 75 mL. The mixture was then subjected to centrifugation (5,000 × *g* for 10 min) to eliminate insoluble substances, and the resulting supernatant served as the plant fermentation medium based on Zong et al. (2023). For mixed fermentation experiments, a 1:1 composite formulation combining both substrates according to Cai et al. (2024).

### 2.2 CTL fermentation

Two CTLs, Dexue No. 1 (Shifang City, Sichuan Province) and Chuxue No. 14 (Enshi City, Hubei Province), were used in this study. The used CTLs were through the three-layer five point method (taking samples from the top, middle, and bottom three layers and five points (surrounding four points and center) of the stacked CTLs),and then were conducted in a controlled humid environment (35°C–37°C, 85%–90% relative humidity) for 48 h to achieve a target moisture content of 25% ± 1%. Subsequently, the loosened CTLs were thoroughly mixed and spread flat on the table and evenly sprayed with fermentation medium using an electronic sprayer, with a ratio of 1 mL medium and 50 mL pure water per kilogram of CTLs. The CTLs without visible water stains were then rebundled. 50 kg CTLs were transferred into one oak barrel and close the lid. The volume of oak barrels was 225 L. Then the oak barrels containing tobacco leaves were transferred to the fermentation room where they underwent fermentation for 30 days under controlled conditions (temperature: 37°C; relative humidity: 75%–80%). Samples from fermented Enshi/Shifang CTLs treated with *T. aurantialba* SCT-F3 fermented broth, cocoa extract, or mixed fermentation medium were labeled accordingly as C1/D1, C2/D2, and C3/D3 respectively. Uninoculated (only sprayed the same volume of pure water as the fermentation medium) Enshi and Shifang CTLs served as controls (C0 and D0). Before analysis, the fermented CTLs will be frozen at −80°C to guarantee that the microorganisms on the CTLs remain unchanged.

### 2.3 Sensory evaluation

The fermented CTLs were carefully processed to form a homogeneous sample, with a circumference of 47 mm and length of 110 mm. And then these cigars should be balanced moisture for 72 h or more under environmental conditions of 16°C–25°C and relative humidity of 65%–75%. There were 10 professional cigar tasters who use the local cyclic smoking method of YC/T 138 (1998) (Tobacco and tobacco products—the sensory evaluation methods), dividing the evaluation into front, middle, and rear sections. All samples underwent comprehensive evaluation based on four key attributes: aroma (intensity, complexity, and maturity), smoke (sharpness, smoothness, and refinement), aftertaste (sweetness, cleanliness, lingering taste), and combustibility (ignition capability, ash coloration, and ash condensation). Each attribute was scored on a scale from 0 to 9 points, representing varying degrees of strength (Zhang et al., 2024b).

### 2.4 Nonvolatile chemical components analysis

The nonvolatile chemical components of six types of plastid pigments, 18 types of amino acids, and five types of polyphenols in the CTLs were determined by high-performance liquid chromatography (HPLC). Briefly, Took 2.0 g of the CTL sample and added 25 mL of 90% acetone. Performed ultrasonic extraction for 20 min, and filtered the extract into the sample for analysis. The HPLC device (Agilent 1,260, Agilent Technologies, Unites States) equipped with a C_18_ column (4 μm, 3.9 mm × 150 mm, Agilent Technologies, Unites States). The mobile phase was isopropanol. The mobile phase was circulated at a rate of 0.5 mL/min, and the column temperature was 30°C. The concentration of chlorophyll a was determined by reading the absorbance at 428 nm, while for others, it was 448 nm. The standard curves of neoxanthin, violaxanthin, lutein, chlorophyll b, chlorophyll a, and β-carotene were established (YC/T 382, 2010, Determination of plastid pigments-high performance liquid chromatography method). Took 100.0 mg of CTL sample and added 20 mL of 50% methanol, ultrasonic extraction for 20 min, filtered into the sample for analysis. HPLC device was equipped with a C_18_ (5 μm, 4.6 mm × 250 mm, Agilent Technologies, Unites States). The mobile phase A was water/methanol/acetic acid (volume ratio 88:10:2), and the mobile phase B was water/methanol/acetic acid (volume ratio 10:88:2). The procedure was as follows: 0 min, 100% mobile phase A; 16.5 min, 80% mobile phase A and 20% mobile phase B; 30 min, 20% mobile phase A and 80% mobile phase B. The mobile phase was circulated at 1 mL/min and the column temperature was 30°C. The detection wavelength was 340 nm. The standard curve of neochlorogenic acid, chlorogenic acid, cryptochlorogenic acid, scopoletin, and rutin contents were established (YC/T 202, 2006, Tobacco and tobacco products—determination of polyphenols—chlorogenic acid, scopletin and rutin). Took 0.25 mg of CTL sample and added 40 mL of 1.0 mmol/L of sodium acetate, ultrasonic extraction for 30 min. An aliquot of 1 mL of extract was mixed with 1 mL of 10% trichloroacetic acid and centrifuged at 10,000× g for 30 min; then a volume of 0.4 mL was sampled. Samples were measured with a HPLC device equipped with a Hypersil ODS (5 μm, 4.0 mm × 250 mm, Agilent Technologies, United States). The mobile phase A was 27.6 mmol/L of sodium acetate/trimethylamine/tetrahydrofuran (volume ratio 500:0.11:2.5), and the mobile phase B was 80.9 mmol/L of sodium acetate/methanol/acetonitrile (volume ratio 1:2:2). The procedure was as follows: 0 min, 8% mobile phase B; 17 min 50% mobile phase B; 20.1 min, 100% mobile phase B; 24 min, 0% mobile phase B. The mobile phase was circulated at 1 mL/min and the column temperature was 40°C. The concentrations of amino acids, except proline (Pro), were determined by reading absorbance at 338 nm, while that of Pro was 262 nm (Zhang et al., 2021).

### 2.5 Metagenomic analysis

The samples (10.0 g) were triturated using liquid nitrogen in a sterile triturator, followed by transfer into 250 mL of sterile phosphate buffer saline and shaking at 4°C for 2 h at 200 r/min. Subsequently, centrifuge at 4°C (12,000 × *g*, 30 min), discard the supernatant, and the resulting precipitate was the surface microbiome of CTLs. Shanghai Majorbio Bio-pharm Technology (Shanghai, China) performed the extraction and fragmentation of total genomic DNA from each sample, construction of PE libraries, and Illumina sequencing. The original sequences underwent quality control to remove *Nicotiana tabacum* genomic DNA (using the Fastp0.20.0 and BWA0.7.9a). Then, the optimized sequences were spliced (using the Megahit1.1.2), and compared against the non-redundant protein sequence (NR) database, Kyoto Encyclopedia of Genes and Genomes (KEGG) database, and Cluster of Orthologous Groups of proteins (COG) database to obtain species and function annotations (similarity >97%).

### 2.6 Statistical analysis

The fermentation experiment was carried out three times, and each experiment had three replicates. The data of sensory evaluation was analyzed through one-way analysis of variance (ANOVA) and Duncan’s multiple comparison test (p < 0.05), using SPSS version 19 software (SPSS Inc., Unites States). Heatmaps and cluster analyses of chemical components, microbial phyla and genera were per-formed using TBtools with row scaled (Chen et al., 2023). Microbial OTUs were analyzed through principal coordinate analysis (PCoA) based on weighted UniFrac distance. Spearman’s correlation coefficient (*p* < 0.001) was used to establish a microbial symbiosis model. Metabolic pathway differences between C-group and D-group CTLs were analyzed using iPath 2.0 (http://pathways.embl.de). SIMCA-P software version 13.0 enabled a partial least-squares (PLS) analysis to determine the contributions of chemical constituents to sensory evaluation, as well as the influence of microbes on chemical constituents. The data of chemical constituents, sensory evaluation, and genus were scaled by unit variance not centered (UVN), the standard deviation is computed around zero. Based on factors with VIP values larger than 1.0 Gephi software version 0.9.2 was employed for network visualizations.

## 3 Results

### 3.1 Sensory evaluation profiles

**FIGURE 1:**
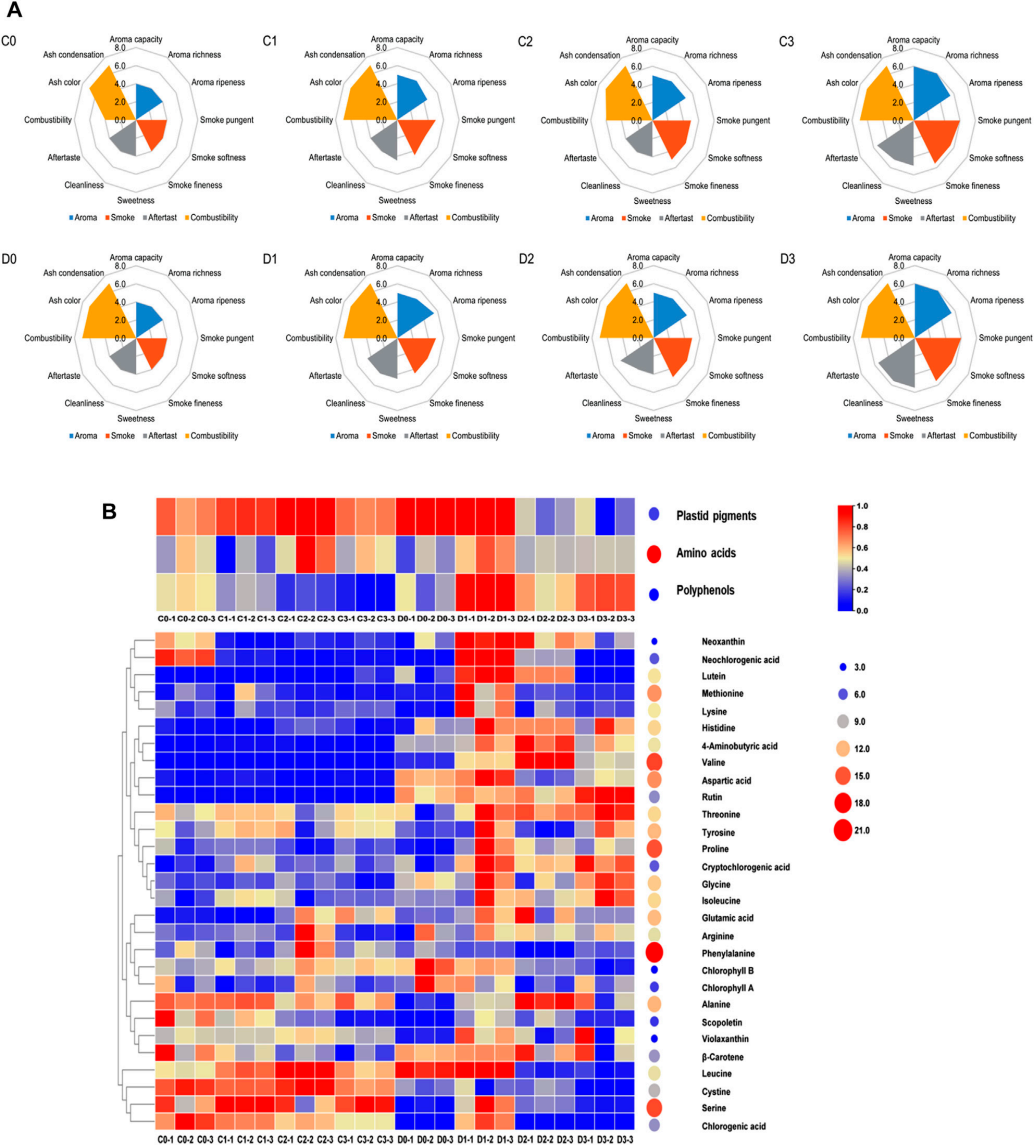
The sensory scores plot and heatmap of nonvolatile chemical components for CTLs. The sensory score plot **(A)** shows the first row from left to right as C0, C1, C2, and C3, and the second row as D0, D1, D2, and D3. The heatmap **(B)** represents the nonvolatile chemical components.

CTLs were fermented in the fermentation room after the addition of fermentation media, and the industrial fermentation conditions remained unchanged from the original ones set when CTLs were initially used (temperature: 37°C; relative humidity: 75%–80%; fermentation time: 30 days). This was performed to verify whether the addition of fermentation media could significantly affect the quality of CTLs and minimize the need for additional equipment investment. After fermentation, the CTLs were prepared into cigars and evaluated by professionally trained evaluation experts. The detailed evaluation scores for the eight CTLs tested are presented in Figure 1A. The scores of CTLs fermented with fermentation media (C1-3 and D1-3) were higher than those of the controls (C0 and D0), and samples treated with a mixed fermentation medium (C3 and D3) demonstrated the highest scores in both the C- and D-groups. Significantly improved CTL qualities (*p* < 0.05) were observed in those subjected to the mixed fermentation medium (C3 and D3) as opposed to the *T. aurantialba* SCT-F3 (C1 and D1) or cocoa (C2 and D2) extracts. In the C-group, fermentation enhanced combustibility, aroma quality, and aroma quantity, whereas smoke concentration increased, irritation decreased, and balance sense improved. In the D-group, an increase in aroma quantity and sweetness was observed, whereas smoke concentration decreased along with irritation.

### 3.2 Overview of the nonvolatile chemical components

The total amino acid content in the CTLs was higher than that of plastid pigments and polyphenols. Phenylalanine (30,190.12–12,110.35 μg/g), serine (2,950.10–538.94 μg/g), and proline (694.00–3,186.41 μg/g) exhibited the highest concentrations in the CTLs. Significant changes were observed in the total content of plastid pigments, amino acids, and polyphenols after fermentation (*p* < 0.05), with variations observed between the C- and D-group samples (Figure 1B). Fermentation reduced the total polyphenol content from 24.52 ± 0.79 to 18.21 ± 2.57 μg/g in C-group CTLs, whereas it increased the total amino acid content from 24,018.41 ± 28.73 to 25,162.42 ± 14.72 μg/g and polyphenol content from 25.54 ± 9.75 to 32.09 ± 5.53 μg/g in D-group CTLs. Specifically, in C-group CTLs, fermentation with specific media resulted in decreased levels of neoxanthin, neochlorogenic acid, and scopoletin; however, lutein exhibited the opposite trend. Conversely, in D-group CTLs, fermentation led to an increase in the concentrations of threonine, alanine, proline, and cryptochlorogenic acid.

### 3.3 Overview of the microbial community

**FIGURE 2:**
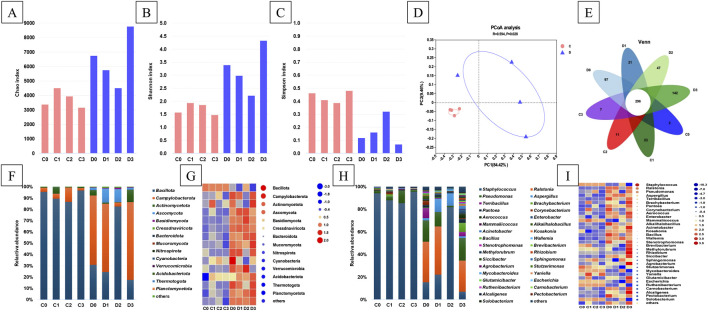
The diversity and relative abundances of microbial communities in CTLs. Alpha diversity was assessed using Chao1 **(A)**, Shannon **(B)**, and Simpson indices **(C)**. Microbial beta diversity **(D)** was measured using weighted UniFrac distance and venn plot **(E)**. Relative abundances **(F)** and heatmap **(G)** at phylum level, relative abundances **(H)** and heatmap **(I)** at genus level.

Raw reads of samples were 4460,6366 to 5916,7570, and optimized reads without host were 2679,6036 to 367,03882. The alpha diversity indices, including the Chao, Shannon, and Simpson indices, of the microbial communities identified in the CTLs are presented in Figures 2A–C. The Chao index of the C-group was lower than that of the D-group, indicating that the total number of microorganisms in the D-group was higher. The addition of a single fermentation medium (C1 and C2) to the C-group increased the total number of microorganisms present, whereas the addition of a mixed fermentation medium (C3) decreased this. The opposite was true for the D-group. The Shannon index of the D-group was also higher, indicating that the microbial community diversity of the D-group was higher than that of C-group. Similar to the Chao index, the addition of a single fermentation medium increased the microbial community diversity in the C-group, whereas a mixed fermentation medium reduced this; the D-group exhibited the opposite trend. These results were consistent with those of the Simpson index. The PCoA results revealed that the microbiota composition explained 93.88% of the microbial variance observed among CTLs (Figure 2D). The C-group CTLs clustered together on the left side of the PCoA plot, whereas the D-group CTLs displayed a relatively more dispersed distribution. This indicates that the different fermentation media used had a more significant impact on the D-group, which was consistent with the aforementioned results. A total of 296 common species were observed across all CTL samples (Figure 2E). Notably, the C-group had fewer unique microorganisms (2–23 operational taxonomic units [OTUs]) than those in the D-group (21–142 OTUs).

### 3.4 Analysis of microbial structure and co-occurrence patterns

The microbial community composition across taxonomic ranks was systematically analyzed using high-throughput sequencing (Figures 2F–I). Among the identified bacterial and fungal phyla, five dominant phyla—Bacillota, Campylobacterota, Actinomycetota, Ascomycota, and Basidiomycota—collectively constituted 99.14%–100.00% of the total microbial phylotypes (Figure 2F). Comparative analysis revealed that the D-group exhibited more pronounced fluctuations in microbial phylum-level composition relative to the C-group. As shown in Figure 2F, the original microbial compositions of CTLs in the C- and D-groups already exhibited significant differences. The C-group was dominated by Bacillota, whereas the D-group consisted of both Bacillota and Campylobacterota. In the C-group, fermentation with single-medium addition reduced the relative abundance of Bacillota, whereas cocoa extract significantly increased the relative abundance of Campylobacterota. In contrast, composite-medium addition resulted in the opposite trend. In the D-group, single-medium treatment increased the relative abundance of Ascomycota, and cocoa extract caused a sharp decrease in the relative abundance of Campylobacterota. Overall, the effects that the same medium had on the different CTLs differed.

The dominant bacterial genera, with relative abundances higher than 5.0% in at least one sample, were *Staphylococcus*, *Ralstonia*, *Pseudomonas*, *Aspergillus*, *Terribacillus*, and *Brachybacterium*. These genera collectively represented between 67.9% and 97.05% of the total abundance measured in each CTL sample (Figure 2H). The changes in microbial genera observed in the D-group were more pronounced than those in the C-group. In C-group CTLs, *Staphylococcus* and *Pseudomonas* emerged as the top two dominant genera, accounting for 91.25%–96.92% of the microbial diversity, ranging from 87.96% to 95.65% and 1.27%–11.16%, respectively. In addition to *Staphylococcus* and *Pseudomonas*, the dominant bacterial genera identified in the D-group included *Ralstonia*, *Aspergillus*, *Terribacillus*, and *Brachybacterium*. The relative abundance of *Aspergillus* was lower in D0 and D3 than in D1 and D2; conversely, the relative abundance of *Terribacillus* showed the opposite trend. Sequencing analysis revealed a higher number of sequences assigned to *Staphylococcus* in C-group CTLs compared with those found in the D-group; however, *Ralstonia*, *Pseudomonas*, *Aspergillus*, *Terribacillus*, and *Brachybacterium* exhibited contrasting trends (Figure 2I). Table 1 presents the taxonomic distributions of the top 25 microbial OTUs based on their relative abundances. Among these, *Staphylococcus* demonstrated the highest representation, constituting 10 of the top 25 most abundant OTUs. Notably, *S*. *nepalensis* emerged as the predominant microorganism in groups C and D2, whereas *R*. *solanacearum* displayed the highest relative abundance in group D, except in D2.

**TABLE 1:** Relative abundances of top 25 microbial species of CTLs.

Taxonomy	Phylum	Species	C0	C1	C2	C3	D0	D1	D2	D3
Bacteria	*Bacillota*	*Staphylococcus nepalensis*	9.2010%	8.5192%	8.6214%	8.5127%	1.3750%	1.4376%	6.0614%	0.5373%
Bacteria	*Campylobacterota*	*Ralstonia solanacearum*	—	—	0.0001%	—	3.2565%	2.6041%	0.0004%	2.0594%
Bacteria	*Bacillota*	*Staphylococcus cohnii*	1.0248%	0.9639%	0.9620%	0.9551%	0.1453%	0.1559%	0.6857%	0.0592%
Bacteria	*Campylobacterota*	*Ralstonia pseudosolanacearum*	—	—	—	—	1.4808%	1.1814%	0.0002%	0.9408%
Bacteria	*Bacillota*	*Staphylococcus* sp. *GDY8P120P*	0.5238%	0.4814%	0.4871%	0.4823%	0.0778%	0.0821%	0.3474%	0.0312%
Bacteria	*Bacillota*	*Staphylococcus xylosus*	0.3286%	0.2668%	0.2793%	0.2668%	0.1047%	0.0787%	0.4591%	0.0313%
Bacteria	*Campylobacterota*	*Pseudomonas fulva*	0.0776%	0.1373%	0.6444%	0.0503%	0.2709%	0.0262%	0.0154%	0.4070%
Bacteria	*Bacillota*	*Staphylococcus saprophyticus*	0.3192%	0.3038%	0.2831%	0.3004%	0.0421%	0.0448%	0.2482%	0.0180%
Bacteria	*Campylobacterota*	*Pseudomonas oryzihabitans*	0.0506%	0.0520%	0.0939%	0.0173%	0.6774%	0.4130%	0.0264%	0.1136%
Bacteria	*Bacillota*	*Staphylococcus equorum*	0.2267%	0.2260%	0.1666%	0.1698%	0.1622%	0.0405%	0.3547%	0.0363%
Eukaryota	*Ascomycota*	*Aspergillus cristatus*	0.0010%	0.0116%	0.0006%	0.0007%	0.1238%	0.5222%	0.5943%	0.0898%
Bacteria	*Bacillota*	*Staphylococcus* sp. *GDY8P131P*	0.2895%	0.2722%	0.2744%	0.2746%	0.0323%	0.0336%	0.1506%	0.0136%
Bacteria	*Bacillota*	*Terribacillus goriensis*	0.0006%	0.0002%	0.0004%	0.0003%	0.7739%	0.0312%	0.0027%	0.2796%
Bacteria	*Campylobacterota*	*Pseudomonas psychrotolerans*	0.0320%	0.0365%	0.0558%	0.0119%	0.5103%	0.2731%	0.0178%	0.0984%
Eukaryota	*Ascomycota*	*Aspergillus chevalieri*	0.0007%	0.0077%	0.0005%	0.0006%	0.0806%	0.3377%	0.3831%	0.0587%
Bacteria	*Bacillota*	*Staphylococcus* sp. *GDH8C109P*	0.1503%	0.1406%	0.1397%	0.1384%	0.0228%	0.0234%	0.0988%	0.0086%
Bacteria	*Bacillota*	unclassified *Staphylococcus*	0.1322%	0.1197%	0.1277%	0.1216%	0.0182%	0.0194%	0.0851%	0.0074%
Bacteria	*Bacillota*	*Terribacillus saccharophilus*	0.0003%	0.0001%	0.0002%	0.0002%	0.4021%	0.0154%	0.0012%	0.1377%
Bacteria	*Actinomycetota*	*Corynebacterium casei*	0.0003%	0.0062%	0.0023%	0.0001%	0.4584%	0.0030%	0.0007%	0.0411%
Bacteria	*Actinomycetota*	unclassified *Ralstonia*	—	—	—	—	0.1996%	0.1572%	—	0.1263%
Bacteria	*Campylobacterota*	*Pseudomonas putida*	0.0143%	0.0219%	0.0991%	0.0089%	0.0507%	0.0136%	0.0106%	0.2531%
Bacteria	*Bacillota*	*Mammaliicoccus sciuri*	0.0744%	0.1125%	0.0680%	0.0642%	0.0165%	0.0100%	0.0434%	0.0334%
Bacteria	*Actinomycetota*	*Brachybacterium paraconglomeratum*	0.0015%	0.0255%	0.0014%	0.0010%	0.0198%	0.0179%	0.0018%	0.3370%
Bacteria	*Bacillota*	*Staphylococcus aureus*	0.0841%	0.0734%	0.0853%	0.0764%	0.0087%	0.0084%	0.0499%	0.0047%
Bacteria	*Campylobacterota*	*Pseudomonas* sp. URMO17WK12:I11	0.0185%	0.0332%	0.1545%	0.0121%	0.0579%	0.0050%	0.0034%	0.0915%

Note: - meant not detected.

**FIGURE 3:**
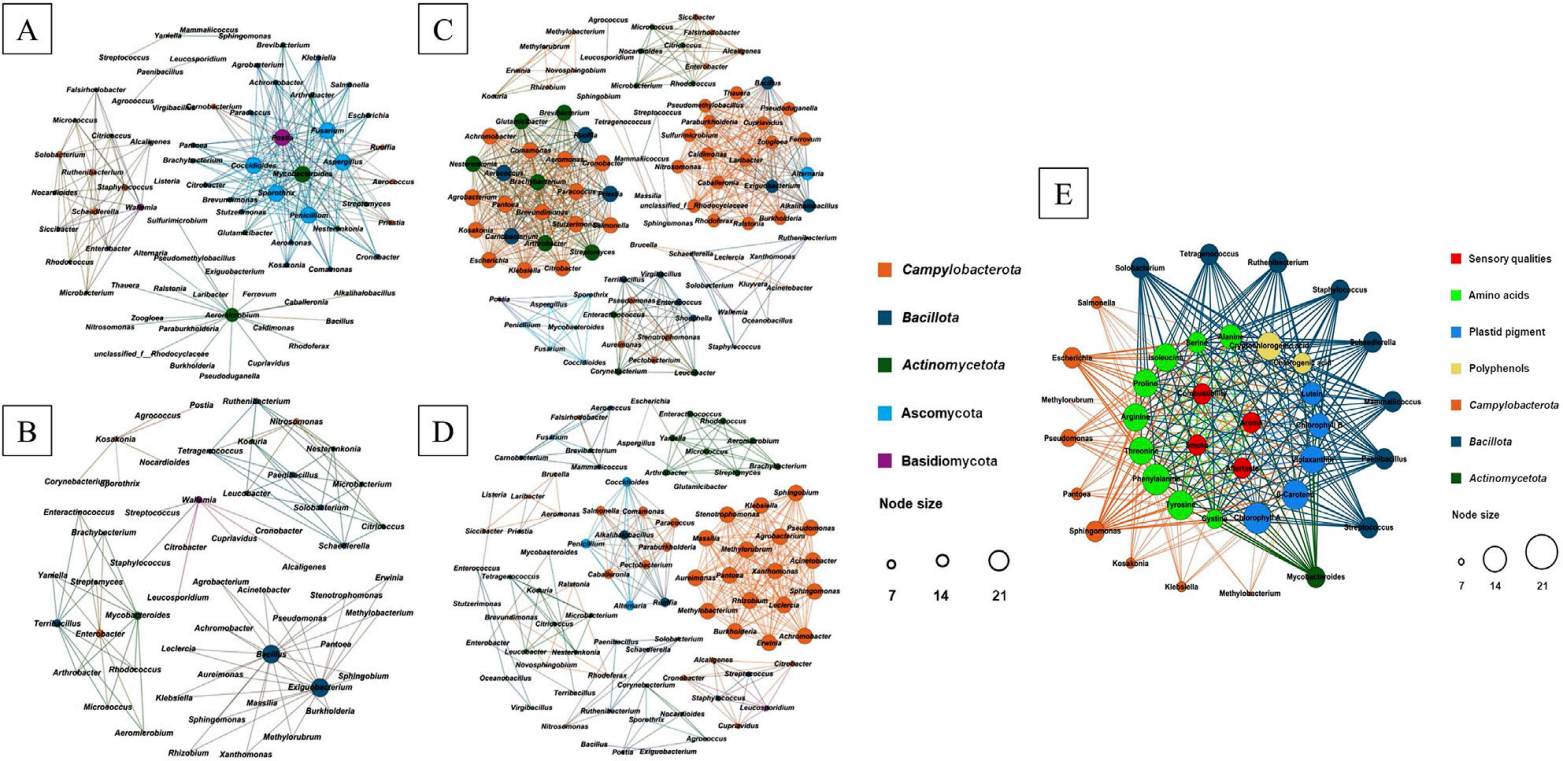
Microbial co-occurrence patterns and correlations between nonvolatile chemical components, microbes, and sensory evaluation of CTLs. Positive **(A)** and negative **(B)** patterns were observed for Enshi CTLs. Positive **(C)** and negative **(D)** patterns were found for Shifang CTLs. Contributions of non-volatile chemical components and microbes to sensory evaluation **(E)**.

The co-occurrence patterns of microorganisms in C- and D-group CTLs were investigated based on the strong and significant correlations observed (*p* < 0.001). In C-group CTLs, 88 nodes and 334 edges with positive correlations and 56 nodes and 102 edges with negative correlations were identified (Figures 3A,B). Among these, 18 hubs (genera with high connectivity, degree ≥15) with positive correlations were observed, including *Pseudomonas*, *Pantoea*, *Acinetobacter*, *Stenotrophomonas*, *Methylorubrum*, *Rhizobium*, *Sphingomonas*, *Agrobacterium*, *Leclercia*, *Burkholderia*, *Aureimonas*, *Methylobacterium*, *Klebsiella*, *Xanthomonas*, *Massilia*, *Achromobacter*, *Erwinia*, and *Sphingobium*, which belong to Campylobacterota and accounted for 45.8% of the targeted to total edges. *Bacillus* and *Exiguobacterium* from Bacillota showed a negative correlation with the aforementioned hubs. In D-group CTLs, 96 nodes and 616 edges with positive correlations and 75 nodes and 238 edges with negative correlations were found (Figures 3C,D). The 44 hubs (highly connected genera, degree ≥15) with positive correlations identified belonged to Campylobacterota (30), Bacillota (7), Actinomycetota (6), and Ascomycota (1), accounting for 75.6% of the targeted to total edges. *Aspergillus*, *Mycobacteroides*, *Penicillium*, *Aeromicrobium*, *Fusarium*, *Coccidioides*, *Sporothrix*, and *Postia* (from Ascomycota, Bacillota, and Basidiomycota) showed a negative correlation with these 44 hubs.

### 3.5 Overview of microbial metabolism

**FIGURE 4:**
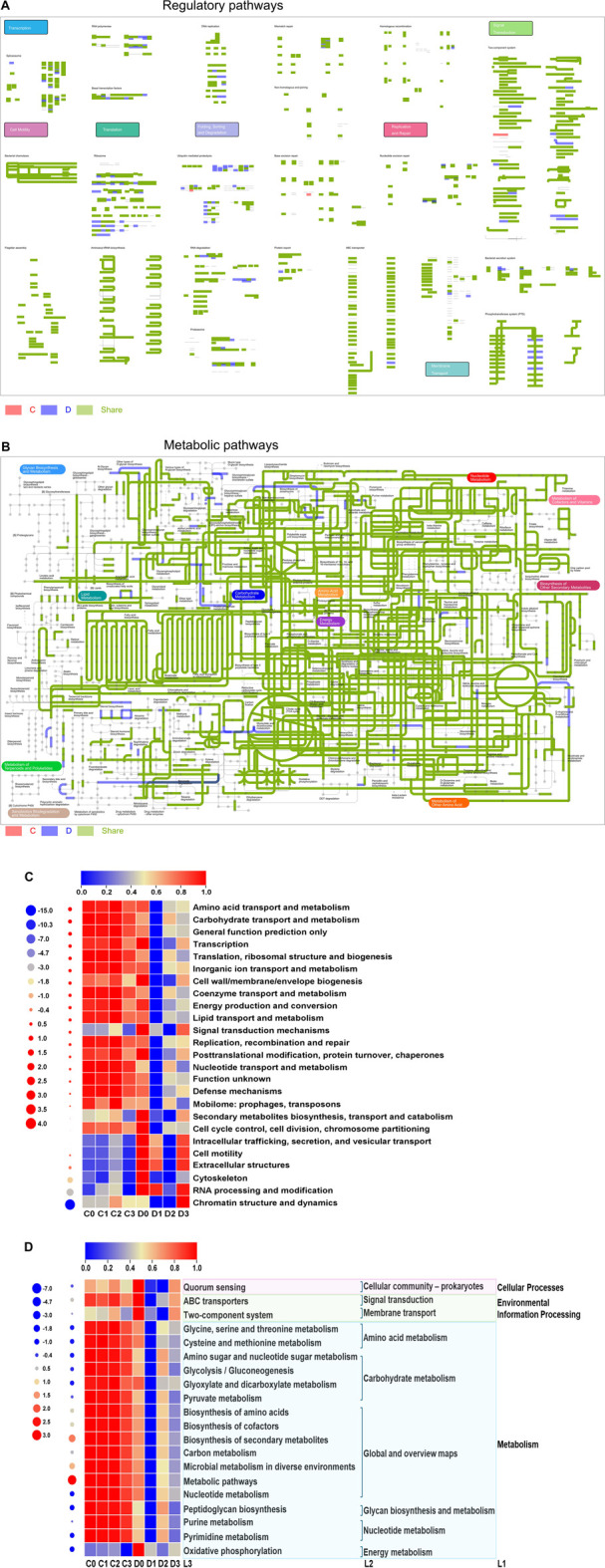
Regulatory pathway **(A)** and metabolic pathway **(B)**, heatmaps of functional gene sequences based on COG **(C)** and KEGG **(D)**.

Figures 4A,B reflected the overall common regulatory pathway and metabolic pathway and their respective pathways of C-group and D-group. A total of 9,624 KOs were identified in the CTLs, with C- and D-group CTLs sharing 8,025 KOs. In the C-group, 63 unique KOs were related to signal transduction, folding, sorting, and degradation. The D-group exhibited 1,537 unique KOs involved in translation, membrane transport, and transcription, as well as terpenoid and polyketide, carbohydrate, and amino acid metabolism (Figures 4A,B).

Figures 4C,D show that the number of functional gene sequences measured was significantly higher in C-group CTLs than in D-group CTLs. Additionally, microbial functional differences between the CTLs before and after fermentation were smaller in the C-group than in the D-group. Functional annotation results of the microbial COG are presented in Figure 4C, where amino acid transport and metabolism, along with carbohydrate transport and metabolism, were ranked among the top three COG functions. KEGG level 1 analysis revealed that microbial functions within the CTLs primarily encompassed metabolism, environmental information processing, and cellular processes. Notably, global overview maps and carbohydrate and amino acid metabolism emerged as the top three pathways within the metabolic processes (Figure 4D).

### 3.6 Contributions of nonvolatile chemical components and microbes to sensory evaluation

Network visualization (Figure 3E) was performed using a two-step PLS analysis approach. In the first step, scaled values of chemical components and of sensory evaluation were used as X- and Y-variables, respectively, to establish correlations. Two significant principal components that accounted for most of the variance in the data matrix were extracted. The cumulative R2X (0.913), R2Y (0.983), and Q2 (0.955) values indicated the excellent suitability of the PLS model for this study. Among these components, 16 compounds belonging to amino acids (nine), plastid pigments (five), and polyphenols (two) exhibited VIP scores >1.0 in both the first and second most significant principal components, demonstrating a positive correlation with all four sensory attributes.

In the second step of the PLS analysis, the scaled values of microbes and of chemical components were employed as X- and Y-variables, respectively, to establish correlations. Two significant principal components were extracted to explain most of the variance observed in the data matrix. The cumulative R2X (0.717), R2Y (0.809), and Q2 (0.6692) values further confirmed the suitability of the PLS model for this study. Among these components, 18 genera belonging to Campylobacterota (nine genera), Bacillota (eight genera), and Actinomycetota (one genus) exhibited VIP scores >1.0 in both the primary and secondary principal components, indicating a positive correlation with the 16 chemical compounds. Except for *Salmonella*, *Methylorubrum*, *Pseudomonas*, *Pantoea*, *Kosakonia*, *Klebsiella*, and *Methylobacterium*, the other 11 genera showed positive correlations with these 16 chemical compounds. Chlorophyll *a* and phenylalanine demonstrated positive correlations with all 16 genera, whereas tyrosine and *β*-carotene only correlated positively with 15 out of the 16 genera, excluding *Methylorubrum*.

### 3.7 Phenylalanine, serine, and proline metabolism and their metabolic pathways

**FIGURE 5:**
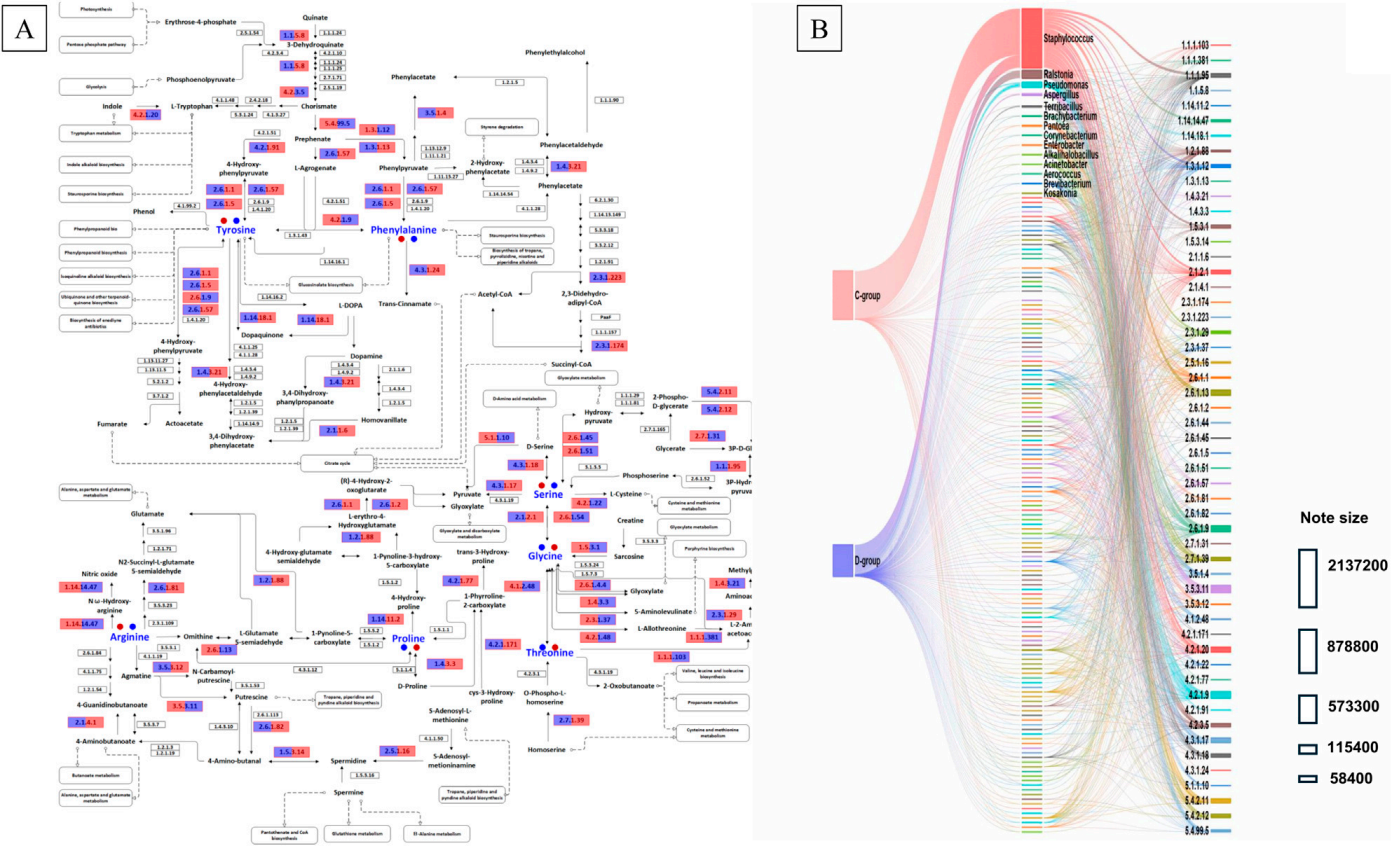
Metabolic pathways related to phenylalanine, serine, and proline metabolism/biosynthesis **(A)**, based on Encyclopedia of Genesand Genomes (KEGG). Microbial sources of key enzymes and their distribution in Enshi and Shifang CTLs **(B)**.

In this study, phenylalanine, serine, and proline constituted the majority of the overall amino acid composition in CTLs, with notable alterations observed in the amino acid profiles of CTLs pre- and post-fermentation. Consequently, the metabolic network pertaining to these three amino acids in CTLs was analyzed. A metabolic network encompassing phenylalanine, serine, and proline metabolism and biosynthesis (Ko00260, Ko00330, Ko00350, Ko00360, and Ko00400), as per KEGG (https://www.kegg.jp/), was constructed based on the annotated enzymes and their corresponding metabolic pathways (Figure 5A). A total of 54 enzymes exhibited significant differences between the C- and D-groups, with 1,247,842 and 574,704 sequences counted, respectively. Among these enzymes in the C-group, 19 enzymes (total count: 865,132 sequences) showed a higher abundance compared with those in the D-group; whereas in the D-group, 35 enzymes (total count: 147,500 sequences) exhibiting an opposite trend. The top five most abundant enzymes included EC:3.5.3.11 (SpeB, agmatinase), EC:4.2.1.9 (ILVD, dihydroxy-acid dehydratase), EC:2.6.1.9 (HisC, histidinol-phosphate aminotransferase), EC:2.6.1.13 (OAT, ornithine aminotransferase), and EC:4.2.1.20 (TRP, tryptophan synthase).

The phenylalanine content in the C-group was higher than that in the D-group, which was attributed to the elevated levels of ILVD and lower levels of EC 4.3.1.24 (PAL, phenylalanine ammonia lyase). Specifically, ILVD facilitates the conversion of l-arogenate to phenylalanine, whereas PAL catalyzes the conversion of phenylalanine to trans-cinnamate. Additionally, higher levels of EC:2.6.1.45 (serine-glyoxylate transaminase), EC:2.6.1.51 (serine-pyruvate transaminase), and EC:2.6.1.54 (pyridoxamine-phosphate transaminase) were measured, whereas lower contents of EC:4.3.1.18 (DSD1, d-serine ammonia-lyase), EC:4.3.1.17 (sdaA, sdaB, tdcG, l-serine dehydratase), and EC:2.1.2.1 (GLYA, SHMT, glycine hydroxymethyltransferase) were observed in the C-group compared with those in the D-group, resulting higher contents of serine in the C-group due to the catalysis of d-serine, hydroxy-pyruvate and glycine into serine, and lower contents of serine to pyruvate action. In the context of proline metabolism, the D-group exhibited a higher abundance of enzymes associated with proline metabolism (EC:2.6.1.13) than the C-group did, resulting in an elevated proline content in the D-group relative to the C-group.

Further analysis of the enzyme source (Figure 5B) revealed that *Staphylococcus*, *Ralstonia*, *Pseudomonas*, *Aspergillus*, and *Terribacillus* were the predominant hosts of genes involved in phenylalanine, serine, and proline metabolic pathways, accounting for 73.54%, 9.45%, 5.91%, 1.93%, and 1.54% of the total genes, respectively. The contribution of microorganisms to each enzyme in the C- and D-groups was calculated based on a normalized enzyme content of 100%. Among them, the phenylalanine-related enzyme, ILVD, in the C-group was mainly derived from *Staphylococcus* and *Pseudomonas*, whereas in the D-group it was mainly derived from *Staphylococcus*, *Pseudomonas*, and *Ralstonia*. In the D-group, another enzyme related to phenylalanine metabolism mainly originated from *Aspergillus*. In the C-group, the six enzymes related to serine mainly originated from *Micrococcus*, *Methylorubrum*, and *Staphylococcus*. *Staphylococcus* was involved in three of the enzymes identified, and the microbial source of each enzyme accounted for over 50%. OAT, which was related to proline, mainly originated from *Staphylococcus* in the C-group, and *Ralstonia* was present in the D-group in addition to *Staphylococcus*. Overall, the *Staphylococcus* genus likely plays an important role in the metabolic network of these three amino acids.

## 4 Discussion

In this study, we used three distinct fermentation media to ferment CTLs obtained from two production areas (Shifang and Enshi Cities). The addition of fermentation medium significantly enhanced the quality of CTLs from Shifang and Enshi, with inter-area differences exceeding those between fermentation media. Post-fermentation, the CTLs exhibited increased aroma quantity and reduced irritancy. Specifically, Enshi CTLs demonstrated improved combustion properties and aroma quality, alongside elevated smoke concentration and enhanced balance. In contrast, Shifang CTLs showed reduced smoke concentration. Fermentation induced chlorophyll degradation and carotenoid accumulation, leading to intensified coloration in Enshi CTLs. Regarding microbial diversity, Enshi CTLs exhibited greater microbial community stability with lesser fermentation impacts, whereas Shifang CTLs displayed more complex microbial networks and significant post-fermentation alterations. Among the six dominant microbes identified, only *Staphylococcus* was more abundant in Enshi CTLs than in Shifang CTLs, whereas *Ralstonia*, *Pseudomonas*, *Aspergillus*, *Terribacillus*, and *Brachybacterium* exhibited the opposite trend. The *Staphylococcus* spp. was associated with protease and lipase activities, which may influence free amino acid levels through multispecies synergistic effects, though not linearly correlated with their abundance. Overall, both fermentation medium and production area characteristics jointly influenced CTLs quality.

The effect that the fermentation medium has on the nonvolatile constituents of CTL fermentation varied across different regions. In the Enshi CTLs, fermentation increased lutein content, while reducing neoxanthin levels of Shifang CTLs. Studies had shown that plastid pigments had an impact on the color of CTLs (Zhang et al., 2020; Wu et al., 2020). And polyphenols and amino acids were related to smoke concentration, aroma quantity and quality, as well as smoke irritancy. Excessive amounts might increase irritancy and pungent taste, causing choking and coughing, whereas insufficient amounts could decrease aroma quantity and quality (Zhao et al., 2007). Although we found that fermentation had an impact on the content of plastid pigments, the optimal levels of polyphenols and amino acids for CTLs have yet to be established.

Microbial community analysis revealed distinct ecological patterns between the Enshi and Shifang CTLs. The alpha diversity indices, PCoA, Venn diagram, and microbial structure plot revealed that the total microbial abundance and diversity of CTLs in Shifang were higher than those in Enshi, with fermentation media exerting a more pronounced influence on Shifang CTLs. This disparity may arise from the inherently simpler microbial composition in Enshi CTLs, characterized by a less diverse core microbiota dominated by fewer operational OTUs. Consequently, alterations to the fermentation substrate predominantly affect the dominant microbial taxa, thereby directly impacting the overall microbial community structure. In contrast, Shifang CTLs exhibited a more complex microbial community with enhanced interspecies interactions. Modifications to a single dominant microorganism within this network could indirectly perturb other coexisting taxa through competitive or cooperative relationships, ultimately inducing structural reorganization of the microbial consortium. This ecological complexity is reflected in the more dispersed PCoA clustering observed for Shifang samples, suggesting greater microbial community variability compared to Enshi. Despite the influence of fermentation, Bacillota, Campylobacterota, Actinomycetota, Ascomycota, and Basidiomycota remained the dominant phyla in these communities, which is consistent with the results of previous studies (Zheng et al., 2022; Zhang et al., 2024a; Wang et al., 2024). Among the six dominant microbial genera examined, only *Staphylococcus* exhibited a higher abundance in Enshi CTLs relative to Shifang CTLs. And the strains, *S. nepalensis*, *S. cohnii*, *S. xylosus*, *S. saprophyticus*, and *S. equorum*, are commonly used as starter cultures in high-salt fermented foods, such as fish sauce (Ma et al., 2022), shrimp paste (Yu et al., 2022), sausage (Yang et al., 2023a), Chinese bacon (Yang et al., 2023b), and French smear-ripened cheese (Irlinger et al., 2012). These strains exhibited protease, lipase, or nitrate reductase activities that contributed to enhanced sensory properties, reduced levels of biogenic amines and bitter amino acids, and the production of flavor compounds, including alcohols, organic acids, and free amino acids. Among them, free amino acids had a significant impact on the sensory quality of cigars (Huang et al., 2023), but the measured content of free amino acids in CTLs was not linearly correlated with the relative abundance of these *Staphylococcus* strains, indicating that the free amino acids present in CTLs might be the result of the joint influence of multiple species. Additionally, *Corynebacterium casei* and *Mammaliicoccus sciuri* have been reported in cheese (Bockelmann et al., 2005) and sausages (Zheng et al., 2024), respectively; these microorganisms are capable of producing organic and amino acids that enhance flavor.

The *R. pseudosolanacearum* and *R. solanacearum* species are pathogens that cause bacterial wilt in tobacco, leading to significant economic losses in tobacco production (Wang et al., 2023; Yang et al., 2022). In the present study, *R. pseudosolanacearum* and *R. solanacearum* were detected only in CTLs obtained from Shifang City, and their abundance decreased after fermentation (Table 1). Among the evaluated treatments, cocoa extract exhibited the most pronounced inhibitory efficacy, with the composite medium showing secondary suppressive effects. This observation suggests that cocoa extract may exert bactericidal effects through direct interactions with *R. pseudosolanacearum* and *R*. *solanacearum*. Therefore, it is crucial to implement preventive measures against bacterial wilt during the cultivation of CTLs in Shifang City, and supplementation with cocoa extract during fermentation processes could serve as an effective strategy for mitigating disease incidence and associated crop damage Moreover, *P. psychrotolerans* exhibited a higher abundance in Shifang CTLs than in Enshi CTLs, but this decreased after fermentation had completed, indicating that medium-based fermentation could reduce the risk of bacterial leaf spot disease in tobacco plants (Li et al., 2023). The *A. chevalieri* and *A. cristatus* species have been identified as the predominant fungal species in dark tea (Hu et al., 2021) and in CTLs, exhibiting cellulase, protease, and polyphenol oxidase activities that potentially facilitate the release of soluble sugars, polysaccharides, amino acids, and phenylacetaldehyde. Notably, CTLs from Shifang City exhibited a higher abundance of *A. chevalieri* and *A. cristatus* than that in CTLs from Enshi City, which could account for their elevated polyphenolic content. Consequently, special attention should be given to mold prevention strategies for CTLs originating from Shifang City. Prevention and control measures should be implemented for the growth environment, transportation, and storage of CTLs. For instance, spraying relevant biological agents in the field can inhibit mold growth in the soil, thereby reducing the initial contamination of CTLs with mold-causing microorganisms. Additionally, attention should be paid to moisture control during transportation and storage because molds require a suitable environment to thrive. Additionally, *C. casei* and *Mammaliicoccus sciuri* have been reported in cheese (Bockelmann et al., 2005) and sausages (Zheng et al., 2024), respectively; these microorganisms are capable of producing organic and amino acids that enhance flavor.

Fermentation not only altered the microbial community but also affected microbial metabolism. The degree of metabolic change in Enshi CTLs was lower than that in Shifang CTLs, and the effects less pronounced than those exhibited by the CTLs themselves, which was consistent with the changes in community structure observed. The microbes present in Shifang CTLs exhibited a greater number of metabolic pathways than those found in Enshi CTLs. Carbohydrate and amino acid metabolism were the primary pathways utilized by microbes within the CTLs, which is consistent with the findings of previous research (Zhang et al., 2023b). Contribution analyses revealed that 18 microbes facilitated the production or transformation of 16 nonvolatile chemical components, ultimately improving sensory qualities of the CTLs. Among these compounds, phenylalanine, serine, and proline were the most abundant. Phenylalanine and proline are aromatic compounds, whereas l-serine plays an important physiological role in cellular metabolism and serves as a precursor for the synthesis of various amino acids (Zhang et al., 2018). Further analysis revealed that *Staphylococcus* occupied an important position in the metabolic network of these three amino acids in the CTL samples. Although more types of enzymes were found in higher quantities in Shifang CTLs than in Enshi CTLs, the total number of enzymes measured was lower. In contrast to Shifang CTLs, Enshi CTLs exhibited higher levels of ILVD, which was primarily derived from *Staphylococcus* and *Pseudomonas*, and lower levels of phenylalanine ammonia-lyase, resulting in increased phenylalanine content. The higher levels of serine-glyoxylate transaminase, serine-pyruvate transaminase, and pyridoxamine-phosphate transaminase were primarily derived from *Micrococcus* and *Methylorubrum*, along with lower levels of d-serine ammonia-lyase, l-serine dehydratase, and glycine hydroxymethyltransferase, which contributed to the elevated serine content in Enshi CTLs. In terms of proline metabolism, the increased presence of ornithine-oxo-acid transaminase, mainly originating from *Staphylococcus* and *Ralstonia*, in Shifang CTLs resulted in a higher proline content than that observed in Enshi CTLs.

## 5 Conclusion

Different fermentation media exerted a lesser effect on the same CTL when compared with the effect that the same media had on different CTLs. The use of fermentation media altered the nonvolatile components, microbial communities, and metabolic profiles of the CTLs. Specifically, fermentation reduced the total polyphenol, neochlorogenic acid, and scopoletin levels in Enshi CTLs, whereas it increased the total amino acid and polyphenol content, including threonine, alanine, proline, and cryptochlorogenic acid, in Shifang CTLs. The microbial complexity observed in the CTLs obtained from Shifang City was higher than that observed in the CTLs obtained from Enshi City. Among the six dominant microbial genera examined, only *Staphylococcus* was more abundant in Enshi CTLs than in Shifang CTLs, whereas *Ralstonia*, *Pseudomonas*, *Aspergillus*, *Terribacillus*, and *Brachybacterium* showed the opposite trend. A total of 18 genera, including *Staphylococcus* and *Pseudomonas*, contributed to the accumulation of 16 compounds, including nine amino acids, five plastid pigments, and two polyphenols, via carbohydrate and amino acid metabolism. Ultimately, this led to an enhancement of sensory attributes, such as aroma, smoke, aftertaste, and combustibility, in the CTLs. Although Shifang CTLs possessed a larger number of enzymes with an increased abundance than that in Enshi CTLs, the overall enzyme count was lower in Shifang CTLs. Overall, *Staphylococcus* was demonstrated to play a significant role in the metabolism of phenylalanine, serine, and proline.

## Data Availability

The data presented in the study are deposited in the NCBI BioProject repository, accession number PRJNA1247148.

## Abbreviations

CTL, Cigar tobacco leaf; OTU, Operational taxonomic units; HPLC, high-performance liquid chromatography; NR, non-redundant protein sequence; KEGG, Kyoto Encyclopedia of Genes and Genomes; COG, Cluster of Orthologous Groups of proteins; PLS, Partial least-squares; PCoA, Principal coordinate analysis.
