# Supplementary material for: Effects and mechanisms of fermentation media on sensory qualities, nonvolatile components, and microbiota in cigar tobacco leaves
Source: Front Bioeng Biotechnol. 2025 Jun 17;13:1578001. doi: 10.3389/fbioe.2025.1578001 (PMC12209301; doi:10.3389/fbioe.2025.1578001)
Supplement: Supplementary file 1 [file Table1.DOCX]

**Supplementary TABLE 1** Correlation coefficients between microbes and chemical components^†^

| Coefficient values | Varibales | | | | | | | | | | | | | | | | | | |
| --- | --- | --- | --- | --- | --- | --- | --- | --- | --- | --- | --- | --- | --- | --- | --- | --- | --- | --- | --- |
|  | *Escherichia* | *Klebsiella* | *Kosakonia* | *Mammaliicoccus* | *Methylobacterium* | *Methylorubrum* | *Mycobacteroides* | | *Paenibacillus* | *Pantoea* | *Pseudomonas* | *Ruthenibacterium* | *Salmonella* | *Schaedlerella* | *Solobacterium* | *Sphingomonas* | *Staphylococcus* | *Streptococcus* | *Tetragenococcus* |
| Chlorogenic acid | 0.0457 | 0.0079 | 0.0123 | 0.0576 | 0.0109 | 0.0098 | 0.0687 | | 0.0638 | 0.0096 | 0.0237 | 0.0623 | 0.006 | 0.0648 | 0.0662 | 0.0557 | 0.0664 | 0.054 | 0.0563 |
| Cryptochlorogenic acid | 0.0409 | 0.022 | 0.0242 | 0.0422 | 0.0237 | 0.0231 | 0.0463 | | 0.0472 | 0.0231 | 0.0296 | 0.0417 | 0.0212 | 0.0436 | 0.0445 | 0.0447 | 0.0448 | 0.0448 | 0.0413 |
| Lutein | 0.0449 | 0.0121 | 0.0159 | 0.0539 | 0.0148 | 0.0138 | 0.063 | | 0.0597 | 0.0137 | 0.0258 | 0.057 | 0.0105 | 0.0594 | 0.0607 | 0.0532 | 0.0609 | 0.052 | 0.0526 |
| Chlorophyll A | 0.0417 | 0.0205 | 0.023 | 0.0443 | 0.0223 | 0.0216 | 0.0493 | | 0.0494 | 0.0216 | 0.0291 | 0.0444 | 0.0195 | 0.0464 | 0.0474 | 0.0463 | 0.0476 | 0.0462 | 0.0434 |
| Chlorophyll B | 0.0451 | 0.0131 | 0.0168 | 0.0536 | 0.0157 | 0.0147 | 0.0624 | | 0.0594 | 0.0146 | 0.0264 | 0.0565 | 0.0115 | 0.0588 | 0.0601 | 0.0531 | 0.0603 | 0.052 | 0.0523 |
| Violaxanthin | 0.0444 | 0.0163 | 0.0196 | 0.0506 | 0.0187 | 0.0178 | 0.058 | | 0.0563 | 0.0178 | 0.0279 | 0.0524 | 0.015 | 0.0546 | 0.0558 | 0.0512 | 0.0561 | 0.0505 | 0.0495 |
| *β*-Carotene | 0.0445 | 0.0171 | 0.0203 | 0.0503 | 0.0194 | 0.0186 | 0.0574 | | 0.0559 | 0.0185 | 0.0284 | 0.0518 | 0.0158 | 0.0541 | 0.0553 | 0.0511 | 0.0555 | 0.0504 | 0.0491 |
| Alanine | 0.0473 | 0.0110 | 0.0151 | 0.0578 | 0.0139 | 0.0128 | 0.0682 | | 0.0641 | 0.0127 | 0.0261 | 0.0617 | 0.0091 | 0.0643 | 0.0657 | 0.0566 | 0.0659 | 0.0551 | 0.0565 |
| Arginine | 0.0447 | 0.0186 | 0.0216 | 0.0495 | 0.0208 | 0.02 | | 0.0561 | 0.0551 | 0.02 | 0.0293 | 0.0506 | 0.0174 | 0.0528 | 0.054 | 0.0507 | 0.0542 | 0.0502 | 0.0484 |
| Cystine | 0.0464 | 0.0025 | 0.0075 | 0.0619 | 0.0058 | 0.0046 | | 0.0753 | 0.0684 | 0.0044 | 0.0209 | 0.0683 | 0.0002 | 0.071 | 0.0726 | 0.0584 | 0.0727 | 0.056 | 0.0604 |
| Isoleucine | 0.042 | 0.0234 | 0.0256 | 0.0429 | 0.0251 | 0.0244 | | 0.0468 | 0.0479 | 0.0245 | 0.0309 | 0.0421 | 0.0226 | 0.044 | 0.0449 | 0.0457 | 0.0452 | 0.0459 | 0.042 |
| Phenylalanine | 0.0457 | 0.0159 | 0.0194 | 0.0526 | 0.0184 | 0.0175 | | 0.0605 | 0.0584 | 0.0174 | 0.0282 | 0.0547 | 0.0145 | 0.057 | 0.0583 | 0.0529 | 0.0585 | 0.0521 | 0.0514 |
| Proline | 0.043 | 0.0175 | 0.0204 | 0.0479 | 0.0196 | 0.0188 | | 0.0544 | 0.0533 | 0.0188 | 0.028 | 0.0491 | 0.0163 | 0.0512 | 0.0524 | 0.049 | 0.0526 | 0.0484 | 0.0468 |
| Serine | 0.0462 | 0.0064 | 0.011 | 0.0593 | 0.0095 | 0.0083 | | 0.0711 | 0.0656 | 0.0082 | 0.0231 | 0.0644 | 0.0043 | 0.067 | 0.0685 | 0.0568 | 0.0687 | 0.0549 | 0.0579 |
| Threonine | 0.0428 | 0.0204 | 0.023 | 0.0458 | 0.0223 | 0.0216 | | 0.0512 | 0.0511 | 0.0216 | 0.0295 | 0.0461 | 0.0193 | 0.0481 | 0.0492 | 0.0477 | 0.0494 | 0.0475 | 0.0448 |
| Tyrosine | 0.045 | 0.0185 | 0.0216 | 0.05 | 0.0207 | 0.0199 | | 0.0567 | 0.0557 | 0.0198 | 0.0294 | 0.0512 | 0.0172 | 0.0534 | 0.0546 | 0.0511 | 0.0548 | 0.0506 | 0.0489 |

Note: The coefficient is significant (*p < 0.05*).
